# Europium Nanoparticle-Based Lateral Flow Strip Biosensors Combined with Recombinase Polymerase Amplification for Simultaneous Detection of Five Zoonotic Foodborne Pathogens

**DOI:** 10.3390/bios13060652

**Published:** 2023-06-14

**Authors:** Bei Jin, Biao Ma, Qing Mei, Shujuan Xu, Xin Deng, Yi Hong, Jiali Li, Hanyue Xu, Mingzhou Zhang

**Affiliations:** 1 Zhejiang Provincial Key Laboratory of Biometrology and Inspection & Quarantine, China Jiliang University, Hangzhou 310018, China; yieio0219@163.com (B.J.); 16a0701109@cjlu.edu.cn (B.M.); 17854301575@163.com (Q.M.); xushujuan31@163.com (S.X.); 15977306909@163.com (X.D.); 18768152453@163.com (Y.H.); 2 Hangzhou Quickgene Sci-Tech. Co., Ltd., Hangzhou 310018, China; qjc1993@126.com; 3 College of Life Science, China Jiliang University, Hangzhou 310018, China; m19357389472@163.com

**Keywords:** lateral flow strip biosensors, recombinase polymerase amplification, zoonotic foodborne pathogens, fluorescent nanomaterials, multiple detection

## Abstract

The five recognized zoonotic foodborne pathogens, namely, *Listeria monocytogenes*, *Staphylococcus aureus*, *Streptococcus suis*, *Salmonella enterica* and *Escherichia coli O157:H7*, pose a major threat to global health and social–economic development. These pathogenic bacteria can cause human and animal diseases through foodborne transmission and environmental contamination. Rapid and sensitive detection for pathogens is particularly important for the effective prevention of zoonotic infections. In this study, rapid and visual europium nanoparticle (EuNP)-based lateral flow strip biosensors (LFSBs) combined with recombinase polymerase amplification (RPA) were developed for the simultaneous quantitative detection of five foodborne pathogenic bacteria. Multiple T lines were designed in a single test strip for increasing the detection throughput. After optimizing the key parameters, the single-tube amplified reaction was completed within 15 min at 37 °C. The fluorescent strip reader recorded the intensity signals from the lateral flow strip and converted the data into a T/C value for quantification measurement. The sensitivity of the quintuple RPA-EuNP-LFSBs reached a level of 10^1^ CFU/mL. It also exhibited good specificity and there was no cross-reaction with 20 non-target pathogens. In artificial contamination experiments, the recovery rate of the quintuple RPA-EuNP-LFSBs was 90.6–101.6%, and the results were consistent with those of the culture method. In summary, the ultrasensitive bacterial LFSBs described in this study have the potential for widespread application in resource-poor areas. The study also provides insights in respect to multiple detection in the field.

## 1. Introduction

A zoonosis is an infectious disease that has jumped from non-human animals to humans, which is caused by viruses, bacteria, parasites and fungi [1]. The rapid and widespread distribution of zoonotic strains poses a major threat to livestock and human health on a global scale [2]. The risk of zoonotic diseases in humans increases with the consumption of animal products [3]. According to the World Health Organization (WHO) [4], 600 million cases of illness caused by contaminated food are reported worldwide each year, resulting in 420,000 deaths and 33 million people at risk. Since 2000, the global economic cost of zoonotic outbreaks has exceeded USD 10 billion [5]. Zoonotic strains have caused a huge economic burden worldwide, examples include *Listeria monocytogenes* (*L. monocytogenes*) [6], *Streptococcus suis* (*S. suis*) [7], *Staphylococcus aureus* (*S. aureus*) [8], *Salmonella enterica* (*S. enterica*) [9] and *Escherichia coli O157:H7* (*E. coli O157:H7*) [10]. In 2022, the Ministry of Agriculture of the People’s Republic of China (PRC) confirmed that salmonellosis, swine streptococcosis and listeriosis are important zoonotic diseases [11]. The European Union (EU) observed that *S. aureus* and *E. coli O157:H7* were major foodborne zoonotic strains in the 2019–2020 period [12]. The five pathogenic bacteria mentioned above are commonly found in undercooked foods, contaminated animal products and food processing environments. The consumption of contaminated products (such as meat and milk products) or exposure to contaminated environments can cause various human diseases (Table S1) [13,14,15,16,17,18,19,20,21,22,23,24,25,26,27,28,29]. Therefore, rapid detection of these pathogens is required for effective disease diagnosis and biomonitoring.

Traditional detection methods for zoonotic pathogens based on bacteriological, morphological and polymerase chain reaction (PCR) techniques have drawbacks, such as their time-consuming nature, complex preprocessing steps and strict experimental conditions [30]. Biosensor technology is becoming increasingly popular in pathogen detection as an alternative to traditional methods due to its higher specificity, sensitivity and economic feasibility [31]. Lateral flow strip biosensors (LFSBs) are highly efficient biosensors with the advantages of chromatographic separation and immunological recognition, which allow the visualization and quantification of target products [32]. LFSB detection performance largely depends on the performance of the signal nanomaterial [33]. Traditional LFSBs used gold nanoparticles (AuNPs) as labels, but this method was limited by low signal strength and poor quantitative detection [34]. In recent years, to compensate for the disadvantages of AuNPs and improve the sensitivity of LFSBs, several new signaling nanomaterials have been developed, such as up-converting phosphor (UCP), quantum dots (QDs) and europium nanoparticles (EuNPs) [35,36]. Among these, lanthanide chelates are complexes of rare earth lanthanide ion-chelating ligands with unique fluorescence characteristics. The chelates possess unique fluorescence characteristics, which make them preferable to other fluorescent markers. Specifically, they have a longer fluorescence decay time, narrower emission spectrum and larger Stokes shift [37]. In recent studies, it was found that the use of EuNPs considerably enhanced the sensitivity and signal-to-noise ratio of lateral flow immunoassay strips [38]. EuNP-LFSBs have been widely used in clinical diagnosis [39], veterinary drug residues [40], antibiotic detection [41] and zoonotic strain assays [42,43]. To further improve the sensor sensitivity, the strategy of nucleic acid signal amplification is extensively used in sensors. Recombinant polymerase amplification (RPA), developed by Piepenburgin in 2006 [44], is an established method for isothermal nucleic acid amplification. The RPA system relies on three enzymes for nucleic acid amplification, recombinase (UvsX and UvsY), single-strand binding protein (gp32) and strand replacement DNA polymerase (Bsu). The recombinase enzyme directs short oligonucleotide primers, forming filaments that recognize a homologous target sequence. Once the specific site is found, the enzyme opens the double strands to allow for the hybridization of the primer and target sequence. This process is aided by the single-stranded DNA binding protein, which prevents dissociation of the primers. The strand-displacing polymerase copies the DNA by adding bases onto the 3′ end of the primer. The process can be performed at temperatures between 37 °C and 42 °C within 15–20 min [45,46], reducing the requirement for high-precision technologies. Compared to other isothermal amplification methods (Table S2) [47,48,49,50,51,52,53,54,55,56], RPA technology has high sensitivity, specificity and low-instrument dependency.

In the present study, we developed quintuple RPA-EuNP-LFSBs for the rapid detection of *L. monocytogenes*, *S. suis*, *S. aureus*, *S. enterica* and *E. coli O157:H7* from complex samples. The use of a mini automatic nucleic acid extractor (Auto-Pure Mini) for pre-sample processing can significantly reduce the preparation time and improve the nucleic acid purity. In addition, a fluorescent strip reader can be employed to accurately quantify the detection results. To enable the simultaneous detection of five zoonotic strains, we designed five pairs of specific primers that targeted conserved genes of the bacteria. Key parameters were optimized, such as the RPA primer concentration, reaction time and temperature, magnesium ion (Mg^2+^) concentration and selection of the nitrocellulose (NC) membrane material. The proposed method can efficiently detect objects within 20 min (including strip detection) at 37 °C. By constructing five antibody-loaded test lines, the quintuple RPA-EuNP-LFSB method facilitated the simultaneous field detection of five target pathogens with a low detection limit (10^1^ CFU/mL). This method overcomes the limitations of single detection objects and low sensitivity in rapid detection. It effectively enables joint inspection of multiple zoonotic strains in the field and has promising market prospects.

## 2. Materials and Methods

### 2.1. Bacterial Culture Preparation and DNA Extraction

A total of 40 bacterial strains were utilized, including 20 strains of the five target pathogens and 20 strains of non-target pathogens, as presented in Table 1. All strains were sourced from the American Type Culture Collection (ATCC) and the China Medical Culture Collection (CMCC), except for CJ 10102 and CJ 10217, which were obtained from laboratory stock. The standard strains of *S. suis*, *S. aureus* and *L. monocytogenes* were streaked and cultured on tryptic soy agar plates (Hopebio, Qingdao, China). After 16 h of incubation at 37 °C, a single colony was extracted in brain–heart infusion broth (BHI, Thermo Fisher Scientific Inc., Waltham, MA, USA), and cultured at 37 °C for 18 h with shaking (200 rpm). Under the same conditions, the remaining strains were plated on the nutrient agar plate (Hopebio, Qingdao, China) and single colonies were cultured in Luria–Bertani broth (LB, Sangon, Shanghai, China). After incubation at 37 °C for 6 h, bacterial culture suspensions were colonized to solid culture plates for colony counting. The number of colonies counted was multiplied by the dilution factor and divided by the volume of the culture plate to obtain the colony-forming units per milliliter (CFU/mL).

Use of the mini automated nucleic acid extractor (Auto-Pure Mini) as a sample preparation tool simplifies the operational steps while providing efficient and rapid extraction techniques (Figure S1 in Supplementary Materials). The operating mechanism was selective adsorption of the target extract through magnetic beads modified with specific chemical groups, enabling efficient high-throughput DNA extraction. The operation process of the Auto-Pure Mini was based on previous experiments [57]. The extracted DNA was quantified using a spectrophotometer and stored at −20 °C.

### 2.2. Reagents and Apparatus

We prepared carboxytetramethylrhodamine (TAMRA) monoclonal antibody (mAb), carboxy fluorescein (FAM) mAb, tetrachlorofluorescein (TET) mAb, cyanine 5 (Cy5) mAb, and biotin mAb in the laboratory. 2-(N-morpholino) ethanesulfonic acid (MES) was obtained from Yuchun Biological Technology Co., Ltd. (Shanghai, China). Goat anti-mouse polyclonal antibody (pAb), bovine serum albumin (BSA), 1-(3-di-methylaminopropy1)-3-ethylcarbodimide hydrochloride (EDC), N-hydroxysuccinimide (NHS), Tween-20 and glycerol were obtained from Merck & Co., Inc. (Rahway, NJ, USA). Carboxylate-modified EuNPs with a diameter of 200 nm were procured from Shanghai Uni Biotech Ltd. (Shanghai, China). Sample pads, conjugate pads, adsorption pads and backing cards were obtained from Dean Biotechnology Co., Ltd. (Hangzhou, China). The NC membranes, including Millipore 90 (M90), Millipore 180 (M180), Sartorius CN95 and Sartorius CN140, were purchased from Microdetection Biotechnology Co., Ltd. (Nanjing, China).

A mini automatic nucleic acid extractor (Auto-Pure Mini, Allsheng Instruments Co., Ltd., Hangzhou, China) was used for the pre-treatment of food samples. An HPY001 row film integrated machine (Wilfen Automation Equipment Co., Ltd., Haining, China) and a CM2000 guillotine cutter (BioDot, Irbine, CA, USA) were used to prepare test strips. A fluorescent strip reader (Suzhou Helmen Precise Instruments, Suzhou, China) was used to quantify the fluorescent band intensity, and a ML-49 Portable Ultraviolet 365 nm flashlight (Moweal Biotechnology Co., Ltd., Shanghai, China) was used for visual inspection. A homogenizer (Bioprep-24, Allsheng Instruments Co., Ltd., Hangzhou, China) was used for food homogenization.

### 2.3. Preparation of EuNP-mAb

To prepare the anti-digoxin mAb conjugated with EuNPs, we proceeded as follows. Firstly, 2 mg of carboxyl EuNPs (10 mg/mL), 30 µL of EDC (10 mg/mL) and 90 µL of NHS (10 mg/mL) were dissolved in 800 µL of MES (0.05 M, pH 8.2). Then, the solution was activated by slow shaking and left to incubate at room temperature for 30 min. After activation, the excess EDC/NHS was removed by centrifugation at 12,000 rpm for 25 min. The precipitate was dissolved in 1 mL of borate buffer saline (BBS, 0.05 M, pH 8.2), followed by the addition of 2 mL of anti-digoxin mAb (10 µg/mL). The mixture was gently shaken for 2 h at room temperature. On completion of protein coupling, 110 µL of blocking solution (15% BSA) was added and the solution was then rotated at room temperature for 1 h. To separate any unreacted polyclonal antibody and BSA, the EuNP-mAb conjugate was centrifuged twice at 13,000 rpm for 20 min. Finally, the sediment was suspended in 1 mL of a storage solution containing 0.1% BSA (*w*/*v*) and kept at 4 °C.

### 2.4. Primer Design and Assembly of Quintuple RPA-EuNP-LFSBs

Before designing the RPA primers, MegAlign software (LaserGene, DNASTAR Inc, Madison, WI, USA) was used to analyze the conservation of the following five genes: *hlyA* from *L. monocytogenes* (GenBank: HM58959), *nuc* from *S. aureus* (GenBank: EF529607.1), *gdh* from *S. suis* (GenBank: AF229683), *fimY* from *S. enterica* (GenBank: JQ665438.1) and *rfbE* from *E. coli O157:H7* (GenBank: HM58959). The five genes were highly conserved and there was no homologous sequence among them. According to the TwistDx instruction manual, the specific primers of *S. suis* were designed using Primer Premier 6.0 software (Premier Biosoft, San Francisco, CA, USA), while the remaining primers were referenced from previous experiments (Table S3) [57]. All primers were synthesized by Sangon Biotech Co., Ltd. (Shanghai, China).

**Figure 1:**
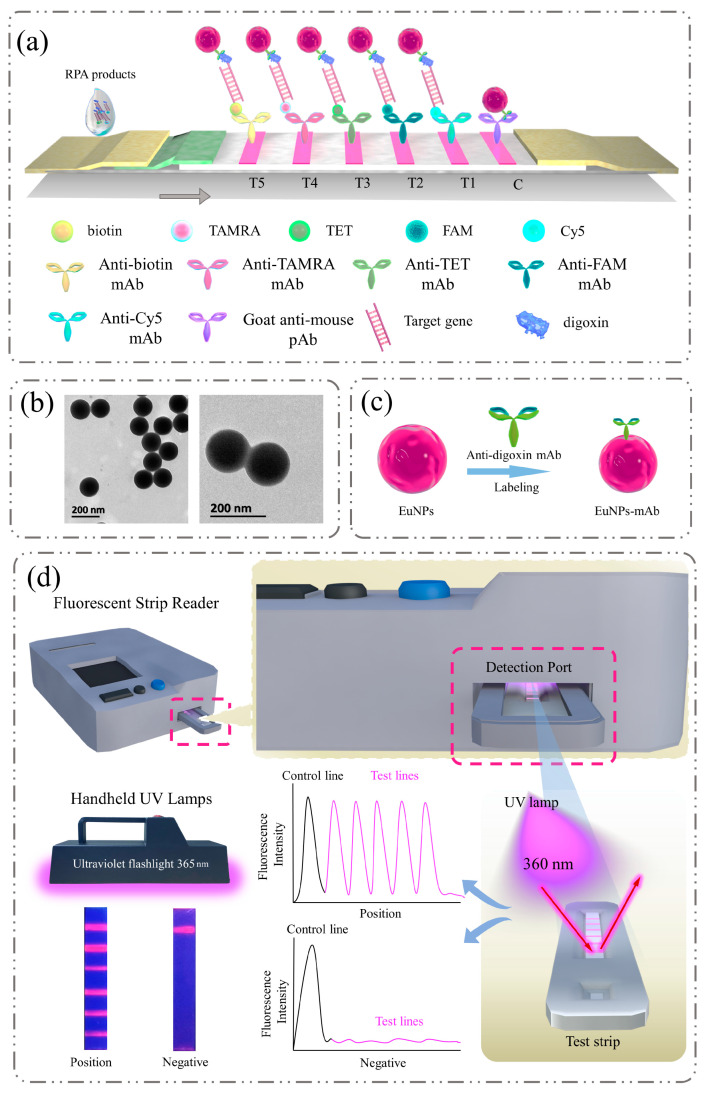
Detection principle of quintuple RPA-EuNP-LFSBs. (**a**) Schematic diagram of detection of amplification products on test strips. “T1”: *L. monocytogenes*; “T2”: *S. aureus*; “T3”: *S. suis*; “T4”: *S. enterica*; “T5”: *E. coli O157:H7*; (**b**) electron microscope images, EuNPs (**left**), EuNPs coupled with digoxin (**right**); (**c**) schematic of the labeling of EuNPs and anti-digoxin; (**d**) visual identification and quantitative analysis of quintuple RPA-EuNP-LFSBs.

The quintuple lateral flow strip consisted of a sample pad, a conjugate pad, NC membranes, an adsorption pad and a backing card. The sample pad and conjugate pad needed to undergo pre-treatment which required soaking them in PBS buffer solution (0.05 M, pH 7.4, containing 1% BSA and 0.05% Tween-20) for 30 min, followed by drying them at a constant temperature of 37 °C for at least 16 h. The prepared EuNP-mAb was evenly distributed onto the conjugate pad (1% BSA, pH 7.4) and the sample pad was wetted with 0.05 M PBS for 30 min. Samples were then dried overnight in a drying oven at 37 °C. As shown in Figure 1a, 2.0 mg/mL goat anti-mouse pAb was immobilized as the C line. Anti-Cy5 mAb, anti-FAM mAb, anti-TET mAb, anti-TAMRA mAb and anti-biotin mAb were immobilized as the T1, T2, T3, T4 and T5 lines, respectively. The distance between the test line (T line) and control line (C line) was 2 mm. Then, the prepared materials were assembled and cut into 2.5 mm wide strips using a strip cutter. Finally, the strips were stored at room temperature below 20% humidity.

### 2.5. Multiplex Reaction Protocols for RPA

The TwistAmp Basic Kit (TwistDX, Cambridge, UK) was used for RPA amplification. The target DNA was prepared as per the above scheme, and sterile water was used as a negative template control (NTC). The final reaction system for the quintuple RPA-EuNP-LFSBs experiment was 50 μL containing 25 μL of 2× reaction buffer, 2 μL of each of the forward primers and reverse primers (10 μM) for the five target pathogenic bacteria and 0.5 μL of each of the templates. The mixture was added to the lyophilized enzyme precipitate and mixed well. Then, 2.5 μL of 14 mM Mg^2+^ was added to the cap of the tube. The RPA reaction was incubated at 37 °C for 25 min. After the reaction was completed, the amplification products were promptly transferred into ice to stop the reaction.

### 2.6. Optimization of Quintuple RPA-EuNP-LFSBs

To achieve optimal performance of the quintuple RPA-EuNP-LFSBs, it was necessary to optimize several key parameters: the primer concentration of RPA, reaction time, reaction temperature, Mg^2+^ concentration and selection of NC membranes. To determine the optimal primer concentration, different concentration gradients ranging from 150 to 450 nM were set. Then, the effectiveness of these concentrations was compared at eight different temperature gradients (33, 34, 35, 36, 37, 38, 39 and 40 °C) and ten different time points (0, 2.5, 5, 7.5, 10, 12.5, 15, 17.5, 20 and 22.5 min) to identify the optimal conditions. After optimizing the initial three conditions, seven different magnesium concentrations (0, 2.8, 5.6, 8.4, 1.2, 14 and 16.8 mM) were evaluated. Four types of membranes with pore sizes and capillary ascent rates were used, with manufacturer-provided rates of fluid flow: CN-140 (134.3 s/40 mm capillary speed down web), CN-95 (96.9 s/40 mm), M-90 (80–100 s/40 mm) and M-180 (175–185 s/40 mm). The material showing the highest fluorescence intensity was selected as the final condition for the test strip.

### 2.7. Sensitivity and Specificity

The sensitivity of quintuple RPA-EuNP-LFSBs was determined using five pathogenic bacteria in the mid-exponential growth phase. The bacterial strains were diluted to 10^0^ CFU/mL, 10^1^ CFU/mL, 10^2^ CFU/mL, 10^3^ CFU/mL, 10^4^ CFU/mL, 10^5^ CFU/mL, 10^6^ CFU/mL, 10^7^ CFU/mL and 10^8^ CFU/mL. The five reference pathogens were mixed at an equal volume concentration level. In the negative control, the DNA template was substituted with an equal volume of double-distilled water.

In the specificity experiment, the quintuple RPA-EuNP-LFSBs was assessed with the DNA extracted from 40 bacterial strains (Table 1). The reaction was performed under the optimal reaction conditions of quintuple RPA-EuNP-LFSBs. Each experiment was performed independently three times, and each test strip was scanned three times.

### 2.8. Artificially Contaminated Food Samples

The samples tested in this study were sourced from local supermarkets in Hangzhou, China, including chicken, pork, beef, lamb, duck and milk. According to the bacteriological analytical manual (BAM) for *L. monocytogenes* [58], *S. aureus* [59], *S. enterica* [60] and *E. coli O157:H7* [61], all food samples were certified as negative for four pathogens. Then, 225 mL of buffered protein water (BPW) was added to each sample (25.0 g ± 0.1 g or 25.0 mL ± 0.1 mL) under sterile conditions to culture *L. monocytogenes*, *S. aureus*, *S. enterica* and *E. coli O157:H7*. According to the national standard for *S. suis* detection [62], the test sample was determined to be negative. As mentioned above, 225 mL of Todd Hewitt broth (THB) was added to the sample under sterile conditions to culture *S. suis*. All samples were homogenized using a homogenizer at 9000 rpm for 2 min. Then, reference bacteria (the IDs of strains: ATCC 19115 for *L. monocytogenes*, ATCC 25923 for *S. aureus*, ATCC 700794 for *S. suis*, ATCC 13076 for *S. enterica* and ATCC 35150 for *E. coli O157:H7*) with concentrations of 10^4^ CFU/mL, 10^3^ CFU/mL, 10^2^ CFU/mL and 10^1^ CFU/mL were added to each sample homogenate. The food samples from each group were extracted using an Auto-Pure Mini, and the obtained DNA was used as the template for RPA. The experiment was divided into two groups. One group was only inoculated with one target strain. Another group was inoculated with five target strains simultaneously. The quintuple RPA-EuNP-LFSB testing was performed under optimal reaction conditions. The fluorescence reader read the fluorescence intensity, and the sample recovery rate was calculated.

### 2.9. Analysis of Quintuple RPA-EuNP-LFSBs in Field Samples

The utility of using quintuple RPA-EuNP-LFSBs as a surveillance tool for detecting *L. monocytogenes*, *S. suis*, *S. aureus*, *S. enterica* and *E. coli O157:H7* in food was assessed. Six types of food samples were randomly purchased from local markets (Hangzhou, China), including chicken, pork, beef, lamb, duck and milk. All food samples were verified as being free of the target pathogenic bacteria. Then, all samples were weighed to 25.0 g ± 0.1 g or 25 mL ± 0.1 mL. Homogenate was prepared in 225 mL BPW or 225 mL THB. The mixture was oscillated at 37 °C and 200 rpm. After incubation for 16 h, 1 mL of enrichment mixture was extracted. The pathogens were determined using quintuple RPA-EuNP-LFSBs and BAM methods.

### 2.10. Statistical Analysis

All measurements were performed in triplicate for each experiment, and all strips were read three times with a fluorescent strip reader. Data were exported through the fluorescent strip reader software (Suzhou Helmen Precise Instruments, Suzhou, China). The T/C value, which is the ratio between the T line and the C line, was calculated using Microsoft Excel software (Microsoft Inc., Redmond, WA, USA).

## 3. Results and Discussion

### 3.1. Assay Principle

The quintuple RPA-EuNP-LFSBs is a membrane-based sensor for detecting *L. monocytogenes*, *S. suis*, *S. aureus*, *S. enterica*, and *E. coli O157:H7* in contaminated food. The principle involved the RPA amplification of target fragments followed by visualization on LFSBs (Figure 1a). The *hlyA* gene of *L. monocytogenes*, *nuc* gene of *S. aureus*, *gdh* gene of *S. suis*, *fimY* gene of *S. enterica* and *rfbE* gene of *E. coli O157:H7* have been used as target genes of the five zoonotic foodborne pathogens in previously reported assays [57,63]. In this study, the five forward primers were labeled with Cy5, FAM, TET, TAMRA and biotin at the 5′ end. All reverse primers were tagged with digoxin (Table S3). After multiplex RPA amplification by using a TwistAmp Basic Kit, five specific products were generated in a single tube: Cy5-digoxin-, FAM-digoxin-, TET-digoxin-, TAMRA-digoxin- and biotin-digoxin-tagged double-stranded DNA. As shown in Figure 1b, EuNPs were functional microspheres with chemical stability and a high lanthanide-specific fluorescence ratio [64]. The EuNPs had the advantages of good stability, high labeling efficiency and sensitivity [40,65]. The bindings of EuNPs and anti-digoxin monoclonal antibody (Figure 1c) were combined with the labeled duplex DNA on the conjugate pad. Then, the conjugates were transported to the NC membrane by capillary force. The different products were captured by anti-Cy5 monoclonal antibody (for detection of *L. monocytogenes* in T1), anti-FAM monoclonal antibody (for detection of *S. aureus* in T2), anti-TET monoclonal antibody (for detection of *S. suis* in T3), anti-TAMRA monoclonal antibody (for detection of *S. enterica* in T4) and anti-biotin monoclonal antibody (for detection of *E. coli O157:H7* in T5), in the five test lines. The remaining EuNP-mAb was immobilized by the anti-mouse polyclonal antibody (pAb) on the control line. For positive samples, the visible test line formed on the NC membrane. Additionally, when there were no amplification products, the C line was always visible. As shown in Figure 1d, the strips can be qualitatively evaluated by the naked eye under a handheld UV lamp (365 nm). Furthermore, a fluorescent strip reader can be used for quantitative measurement. The fluorescence signals of the T and C lines were collected and analyzed using the portable instrumentation. These signals were converted into standard curves based on the T/C value and the contents of the substance.

### 3.2. Optimization of the Quintuple RPA-EuNP-LFSBs

**Figure 2:**
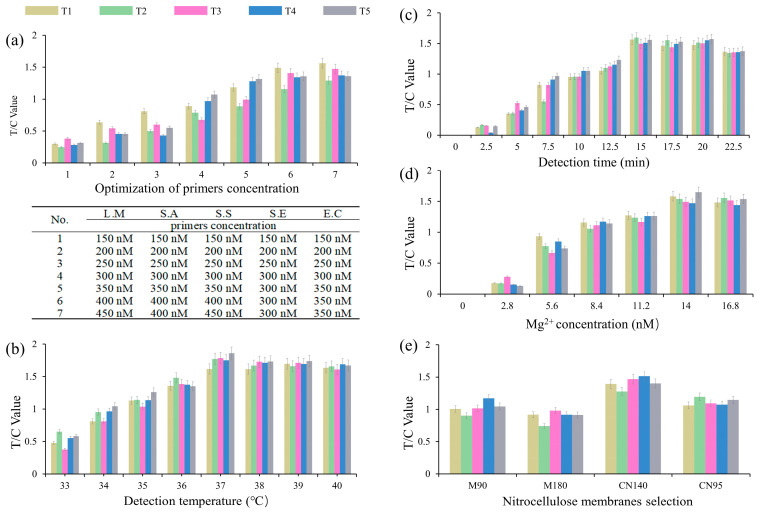
Optimization of the quintuple RPA-EuNP-LFSBs. (**a**) Primer concentration, different ratios of primers are shown in the three-line table; (**b**) detection temperature; (**c**) detection time; (**d**) Mg^2+^ concentration; (**e**) NC membrane selection. Each parameter is shown at the bottom of the bar graph. Three replicates are shown. L.M: *L. monocytogenes*; S.A: *S. aureus*; S.S: *S. suis*; S.E: *S. enterica*; E.C: *E. coli O157:H7*.

RPA primer concentration, reaction time and temperature, Mg^2+^ concentration and the selection of NC membrane material were systematically optimized through a series of experiments, resulting in the achievement of better detection efficiency and sensitivity of the quintuple RPA-EuNP-LFSBs. The concentration of primers was found to be a critical factor affecting the efficiency and specificity of RPA reactions in previous studies [57]. An inadequate primer concentration was shown to reduce the speed and yield of the RPA reaction. Conversely, an excessively high primer concentration resulted in non-specific amplification and primer dimer formation [66]. With reference to previous experiments [57], a single RPA-EuNP-LFSBs of 150 nM was used as the initial primers concentration. The quintuple RPA-EuNP-LFSBs were optimized at 150 nM–450 nM. As shown in Figure 2a, the primer concentration was divided into seven groups, and the T/C values gradually increased as the primer concentration increased. The amplification efficiency reached consistency when the primer concentration was 450 nM for *L. monocytogenes*, 400 nM for *S. aureus*, 450 nM for *S. suis*, 400 nM for *S. enterica* and 400 nM for *E. coli O157:H7*.

In addition, the optimum temperature for the RPA reaction was generally 37–42 °C, and the optimum reaction time was generally between 10–25 min [57]. By optimizing parameters such as temperature and time, the sensitivity and specificity of the RPA technology could be improved to achieve efficient and accurate environmental analysis [48]. As shown in Figure 2b, the best T/C value was found in the temperature range 37–39 °C, and 37 °C, with a lower energy consumption, was selected for subsequent detection, in the eight groups of reaction temperature optimization. Additionally, the reaction temperature could be easily maintained through various methods such as heating, using a water bath, or relying on human body temperature. As shown in Figure 2c, the RPA reaction times of the 10 groups (0–22.5 min) were determined, and there was no significant difference between 15 min and 22.5 min. In order to provide maximum sensitivity while minimizing the measurement time, 15 min was used in the subsequent experiments. The amplification products were detected after incubating at 37 °C for 15 min. To corroborate the optimized reaction conditions, including RPA primer concentration, reaction temperature and time, the results of electrophoresis gel are shown in Figure S2. When conventional PCR is selected to identify pathogenic bacteria, the reactions must be completed in less than 1 h through a specific thermal cycler [30]. The quintuple RPA-EuNP-LFSBs involve an isothermal reaction and do not rely on instrumentation, thereby reducing their operation time compared with PCR-based detection. The concentration of Mg^2+^ in RPA is believed to affect the amplification efficiency, as suggested by previous studies [67,68]. Therefore, optimizing the Mg^2+^ concentration was essential for multiple RPA reactions to occur efficiently. As shown in Figure 2d, five pathogenic bacteria showed superior results in the range 14 mM–16.8 mM, and 14 mM was identified as the optimal concentration of Mg^2+^ for RPA.

Research has indicated that the adsorption capacity of surface antibodies varies depending on the type of NC membrane material used [69]. Different types of NC membranes have different porosity and flow rates, which significantly affected the results of the quintuple RPA-EuNP-LFSBs. Four groups (M90, M180, CN95, CN140) were selected as the NC membranes with different materials to compare the fluorescence intensity. The results are shown in Figure 2e; CN140 has a higher sensitivity and higher fluorescence signal intensity than the other materials.

### 3.3. Sensitivity and Specificity

**Figure 3:**
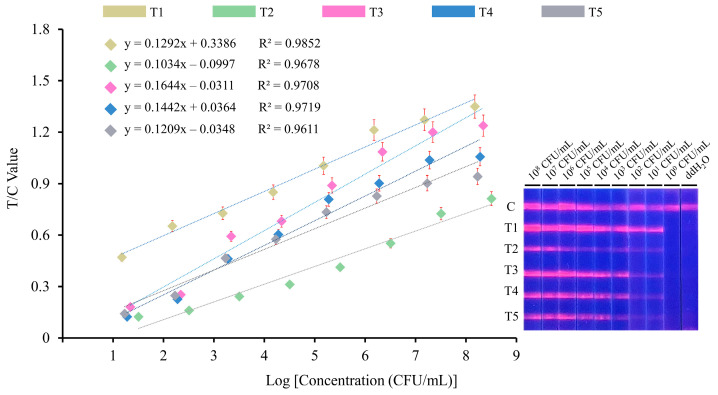
Sensitivity analysis of quintuple RPA-EuNP-LFSBs. The initial concentrations of *L. monocytogenes*, *S. aureus*, *S. suis*, *S. enterica* and *E. coli O157:H7* were 1.5 × 10^8^ CFU/mL, 3.2 × 10^8^ CFU/mL, 2.2 × 10^8^ CFU/mL, 1.9 × 10^8^ CFU/mL and 1.7 × 10^8^ CFU/mL, respectively. The amplified products were observed under a 365 nm UV lamp. The intensity showed linear correlation with the concentration of pure cultures. T1: *L. monocytogenes*; T2: *S. aureus*; T3: *S. suis*; T4: *S. enterica*; T5: *E. coli O157:H7*.

The optimized parameters were used to evaluate the sensitivity of quintuple RPA-EuNP-LFSBs. Five pathogenic bacteria were 10-fold serially diluted from 10^8^ to 10^0^ CFU/mL. The same concentration levels of the five bacterial solutions were mixed together in equal volume. The fluorescence reader was used to quantitate the digital signals of the C and T lines, and the standard curves were established. The experiments were repeated three times. As shown in Figure 3, the T/C value, the ratio of fluorescence signal intensity, increases with a high concentration of template DNA. There were no distinct detection lines when the concentration was below 10^1^ CFU/mL. The visual detection limits were 1.5 × 10^1^ CFU/mL for *L. monocytogenes*, 3.2 × 10^1^ CFU/mL for *S. aureus*, 2.2 × 10^1^ CFU/mL for *S. suis*, 1.9 × 10^1^ CFU/mL for *S. enterica* and 1.7 × 10^1^ CFU/mL for *E. coli O157:H7*. Therefore, the average sensitivity of quintuple RPA-EuNP-LFSBs was 10^1^ CFU/mL. The correlation coefficients (R^2^) for each variable are as follows: R^2^ = 0.9852 for *L. monocytogenes*, R^2^ = 0.9678 for *S. aureus*, R^2^ = 0.9708 for *S. suis*, R^2^ = 0.9719 for *S. enterica*, and R^2^ = 0.9611 for *E. coli O157:H7*.

Biosensors based on nucleic acid amplification (Table S4) [70,71,72,73,74,75,76,77,78] have been applied for the detection of pathogenic bacteria, such as pNC-based strip biosensors, electrochemical biosensors and SERS-based LF strip biosensors. Compared with traditional methods, these biosensors have the advantages of simplicity, sensitivity and specificity. However, they are insufficient when faced with the requirement of multiple targets in the field. In this paper, the quintuple RPA-EuNP-LFSBs provide a multi-objective, highly sensitive, synchronous and rapid detection tool for testing zoonotic foodborne pathogens.

**Figure 4:**
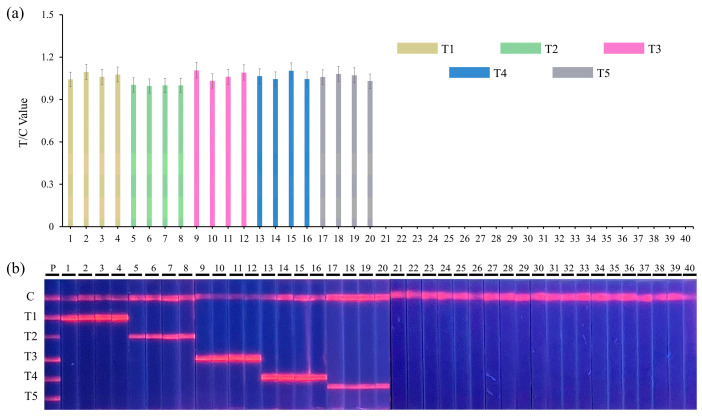
Specific analysis of quintuple RPA-EuNP-LFSBs. (**a**) Result of T/C values data analysis, (**b**) detection of quintuple RPA-EuNP-LFSBs. The numbers 1–40 reference the bacteria in Table 1; P: positive samples; T1: *L. monocytogenes*; T2: *S. aureus*; T3: *S. suis*; T4: *S. enterica*; T5: *E. coli O157:H7*.

The important indexes with which to evaluate the efficiency of the detection methods are specificity and accuracy [77]. A total of 20 bacterial target strains (Table 1), including *L. monocytogenes* (n = 4), *S. aureus* (n = 4), *S. suis* (n = 4), *S. enterica* (n = 4), *E. coli O157:H7* (n = 4) and 20 other non-target pathogens, were used to verify the specificity of quintuple RPA-EuNP-LFSBs. The quantitative results obtained with a fluorescence reader for the five target strains are presented in Figure 4a. The lateral flow strip photograph captured under UV light is shown in Figure 4b. The results indicate that only the first 20 target pathogens showed positive signals in the detection, while the 20 non-target pathogens showed no signal. This indicated that quintuple RPA-EuNP-LFSBs were specific to their corresponding targets.

### 3.4. Detection of Quintuple RPA-EuNP-LFSBs in Artificially Contaminated Food

The quintuple RPA-EuNP-LFSBs successfully identified six types of spiked food samples, i.e., chicken, pork, beef, lamb, duck and milk. To simulate multiple strains in food samples, we prepared a concentration of 1.9 × 10^4^ CFU/mL for *L. monocytogenes*, 3.8 × 10^4^ CFU/mL for *S. aureus*, 2.4 × 10^4^ CFU/mL for *S. suis*, 2.2 × 10^4^ CFU/mL for *S. enterica* and 1.9 × 10^4^ CFU/mL for *E. coli O157:H7*, and diluted them from 10^4^ to 10^1^ CFU/mL. Equal volumes of the same concentration of target bacteria were added into the samples for artificial contamination. The experiments were divided into two groups: single contamination and contamination with five strains. The artificial contamination of the individual strains in the samples, with recovery rates of 91.6 to 101.1% for the spiked samples, is shown in Table S5. The co-existence of the five target bacteria in food samples and their recovery rates in spiked samples of 90.6 to 101.6% are presented in Table S6. Compared to other biosensor detection methods (Table S4), the quintuple RPA-EuNP-LFSBs were able to detect the lowest detection limit (10^1^ CFU/mL) of five bacteria in various food sample matrices. These results demonstrate that the method was able to accurately and consistently differentiate the spiked samples.

### 3.5. Detection of Quintuple RPA-EuNP-LF/SBs in Actual Samples

To further verify the capability and accuracy of quintuple RPA-EuNP-LFSBs, we evaluated 15 food samples including chicken, pork, beef, lamb, duck and milk. All food samples were extracted for genomic DNA and subjected to quintuple RPA-LFIA, BAM or national standard detection, and the results were consistent as shown in Table 2. The positive detection rate of *S. aureus* was 6.7%, the positive detection rate of *E. coli O157:H7* was 13.3%, and the remaining three strains were negative. The quintuple RPA-EuNP-LFSBs demonstrated good performance in actual samples detection, which made them more suitable for field detection or detection in areas with resource shortages. In addition, the quintuple RPA-EuNP-LFSBs were more cost effective, with the estimated cost per reaction of around 12 USD, than the test price of real-time PCR detection kit on the market.

## 4. Conclusions

In this paper, we presented the development of quintuple RPA-EuNP-LFSBs, which utilized europium nanoparticles, recombinant polymerase amplification and a lateral flow strip biosensor. This innovative approach offers rapid DNA extraction, amplification of target genes and the capability to detect five pathogenic bacteria simultaneously. The rapid synchronous amplification and visual judgment of multi-objective results for five zoonotic foodborne pathogens (*Listeria monocytogenes*, *Staphylococcus aureus*, *Streptococcus suis*, *Salmonella enterica* and *Escherichia coli O157:H7*) were successfully conducted via one-tube RPA and demonstrated the capability of detecting multiple targets. The entire process was completed within 20 min (including 5 min for test strip display) at 37 °C using optimized key parameters. The average sensitivity of the quintuple RPA-EuNP-LFSBs reached 10^1^ CFU/mL. The recoveries of the five pathogens ranged from 90.6 to 101.6% in the spiked sample experiments. Furthermore, the actual sample detection results were consistent with those of culture assay. In summary, the proposed quintuple RPA-EuNP-LFSB method was designed for its ease of use and excellent fluorescence performance, enhancing its practicality and availability. It can not only achieve the purpose of simple, sensitive and specific detection, but also provides an effective technical means for the field inspection of multiple zoonotic diseases with good market promotion prospects.

## Figures and Tables

**Table 1:** List of bacteria used in this study.

Sample Number	Species	Serotype	ID of Strains	Quintuple RPA-EuNP-LFSBs Test Results				
				*hlyA*	*nuc*	*gdh*	*fimY*	*rfbE*
1	*Listeria monocytogenes*	4b	ATCC 19115	+	−	−	−	−
2	*Listeria monocytogenes*	4b	ATCC 13932	+	−	−	−	−
3	*Listeria monocytogenes*	1/2a	ATCC 19111	+	−	−	−	−
4	*Listeria monocytogenes*	2	ATCC 19112	+	−	−	−	−
5	*Staphylococcus aureus*		ATCC 25923	−	+	−	−	−
6	*Staphylococcus aureus*		ATCC 35556	−	+	−	−	−
7	*Staphylococcus aureus*	3	CICC 12600	−	+	−	−	−
8	*Staphylococcus aureus*		CICC 21648	−	+	−	−	−
9	*Streptococcus suis*		ATCC 700794	−	−	+	−	−
10	*Streptococcus suis*		ATCC 700796	−	−	+	−	−
11	*Streptococcus suis*		CJ 10102	−	−	+	−	−
12	*Streptococcus suis*		CJ 10217	−	−	+	−	−
13	*Salmonella enterica*	Enteritidis	ATCC 13076	−	−	−	+	−
14	*Salmonella enterica*	Enteritidis	CICC 21513	−	−	−	+	−
15	*Salmonella enterica*	Enteritidis	ATCC 29629	−	−	−	+	−
16	*Salmonella enterica*	Enteritidis	ATCC 29631	−	−	−	+	−
17	*Escherichia coli*	*O157:H7*	ATCC 35150	−	−	−	−	+
18	*Escherichia coli*	*O157:H7*	ATCC 35218	−	−	−	−	+
19	*Escherichia coli*	*O157:H7*	CICC 24187	−	−	−	−	+
20	*Escherichia coli*	*O157:H7*	CICC 21530	−	−	−	−	+
21	*Bacillus coagulans*		CICC 20138	−	−	−	−	−
22	*Bacillus cereus*		ATCC 10876a	−	−	−	−	−
23	*Bacillus cereus*		ATCC 9139	−	−	−	−	−
24	*Bacillus cereus*		CICC 21261	−	−	−	−	−
25	*Bacillus vallismortis*		CICC 21224	−	−	−	−	−
26	*Cronobacter sakazakii*		CICC 24338	−	−	−	−	−
27	*Cronobacter sakazakii*		CICC 24125	−	−	−	−	−
28	*Campylobacter jejuni*		CICC 22936	−	−	−	−	−
29	*Campylobacter jejuni*		ATCC 49349	−	−	−	−	−
30	*Campylobacter jejuni*		CICC 22937	−	−	−	−	−
31	*Clostridium perfringens*		ATCC 13124	−	−	−	−	−
32	*Enterobacter aerogenes*		CICC 10293	−	−	−	−	−
33	*Enterobacter aerogenes*		CICC 10418	−	−	−	−	−
34	*Enterobacter aerogenes*		CICC 20051	−	−	−	−	−
35	*Streptococcus pyogenes*		CICC 10373	−	−	−	−	−
36	*Streptococcus pyogenes*		CICC 10356	−	−	−	−	−
37	*Streptococcus mutans*		CICC 10387	−	−	−	−	−
38	*Shigella flexneri*		CICC 10865	−	−	−	−	−
39	*Shigella flexneri*		CICC 21534	−	−	−	−	−
40	*Shigella sonnei*		CICC 21535	−	−	−	−	−

“+”: positive result; “−”: negative result; CJ 10102 and CJ 10217: from laboratory stock; *hlyA*: *hlyA* gene of *L. monocytogenes*; *nuc: nuc* gene of *S. aureus*; *gdh*: *gdh* gene of *S. suis*; *fimY*: *fimY* gene of *S. aureus*; *rfbE*: *rfbE* gene of *E. coli O157:H7*.

**Table 2:** Comparison of actual samples detected by the quintuple RPA-EuNP-LFSBs and culture method.

Samples	*L. monocytogenes*		*S. aureus*		*S. suis*		*S. enterica*		*E. coli O157:H7*	
	RPA-EuNP-LFSBs	Culture Method ^a^	RPA-EuNP-LFSBs	Culture Method ^a^	RPA-EuNP-LFSBs	Culture Method ^b^	RPA-EuNP-LFSBs	Culture Method ^a^	RPA-EuNP-LFSBs	Culture Method ^a^
Chicken-1	−	−	−	−	−	−	−	−	−	−
Chicken-2	−	−	−	−	−	−	−	−	+	+
Chicken-3	−	−	−	−	−	−	−	−	−	−
Pork-1	−	−	+	+	−	−	−	−	+	+
Pork-2	−	−	−	−	−	−	−	−	−	−
Pork-3	−	−	−	−	−	−	−	−	−	−
Beef-1	−	−	−	−	−	−	−	−	−	−
Beef-2	−	−	−	−	−	−	−	−	−	−
Beef-3	−	−	−	−	−	−	−	−	−	−
Lamb-1	−	−	−	−	−	−	−	−	−	−
Lamb-2	−	−	−	−	−	−	−	−	−	−
Lamb-3	−	−	−	−	−	−	−	−	−	−
Duck-1	−	−	−	−	−	−	−	−	−	−
Duck-2	−	−	−	−	−	−	−	−	−	−
Duck-3	−	−	−	−	−	−	−	−	−	−
Milk-1	−	−	−	−	−	−	−	−	−	−
Milk-2	−	−	−	−	−	−	−	−	−	−
Milk-3	−	−	−	−	−	−	−	−	−	−
Total	−	−	1	1	−	−	−	−	2	2
Positive Detection rate	0%	0%	6.7%	6.7%	0%	0%	0%	0%	13.3%	13.3%

“a”: the bacteriological analytical manual, BAM; “b”: the national standard, GB/T 19915.2-2005; “+”: positive result; “−”: negative result.

## Data Availability

The data presented in this study are available in article.

## Supplementary Materials

The following supporting information can be downloaded at: https://www.mdpi.com/article/10.3390/bios13060652/s1. Figure S1: The operation procedure of the Auto-Pure Mini extractor.; Figure S2: Agarose gel electrophoresis results for optimization of RPA amplification. Table S1: Comments on five types of pathogenic bacterial infection routes and symptoms; Table S2: Characteristics of RPA and other isothermal amplification technologies; Table S3: Sequences of primers used in this study; Table S4: Comparison of quintuple RPA-EuNP-LFSBs with other biosensors; Table S5: Artificial contamination of individual strains in the samples; Table S6: Five target bacteria co-existed in the food samples. References [13,14,15,16,17,18,19,20,21,22,23,24,25,26,27,28,29,47,48,49,50,51,52,53,54,55,56,57,70,71,72,73,74,75,76,77,78] are cited in the supplementary materials.
