# Supplementary material for: Europium Nanoparticle-Based Lateral Flow Strip Biosensors Combined with Recombinase Polymerase Amplification for Simultaneous Detection of Five Zoonotic Foodborne Pathogens
Source: Biosensors (Basel). 2023 Jun 14;13(6):652. doi: 10.3390/bios13060652 (PMC10295953; doi:10.3390/bios13060652)
Supplement: Supplementary file 1 [file biosensors-13-00652-s001.zip › biosensors-2401592-supplementary.pdf]

Supplementary material

Europium Nanoparticle-Based Lateral Flow Strip Biosensors Combined with Recombinases Polymerase Amplification for Simultaneous Detection of Five Zoonotic Foodborne Pathogens

Bei Jin [1,†], Biao Ma [1,†], Qing Mei [1], Shujuan Xu [1], Xin Deng [1], Yi Hong [1], Jiali Li [2], Hanyue Xu [3] and Mingzhou Zhang [1,*]

1 Zhejiang Provincial Key Laboratory of Biometrology and Inspection & Quarantine, China Jiliang University, Hangzhou 310018, China
2 Hangzhou Quickgene Sci-Tech. Co., Ltd., Hangzhou 310018, China
3 College of Life Science, China Jiliang University, Hangzhou 310018
* Correspondence: zmzcjlu@cjlu.edu.cn, 86-571-86914476, Fax: 86-571-86914510.
† These authors have contributed equally to this work.

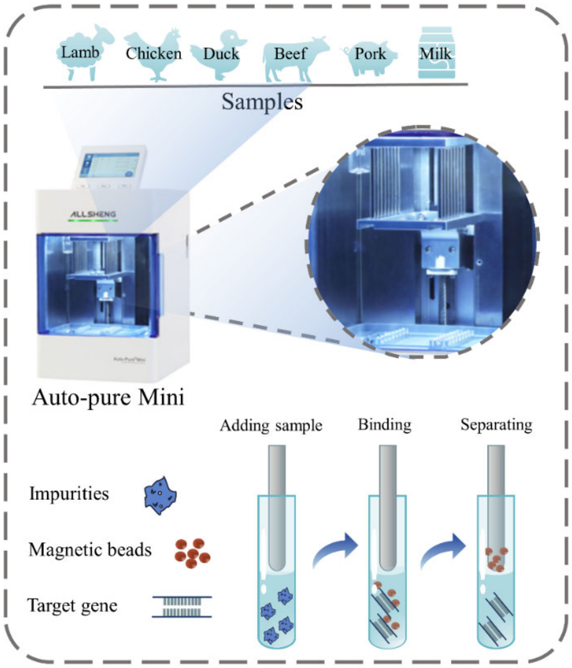

Figure S1. The operation procedure of the Auto-Pure Mini extractor.

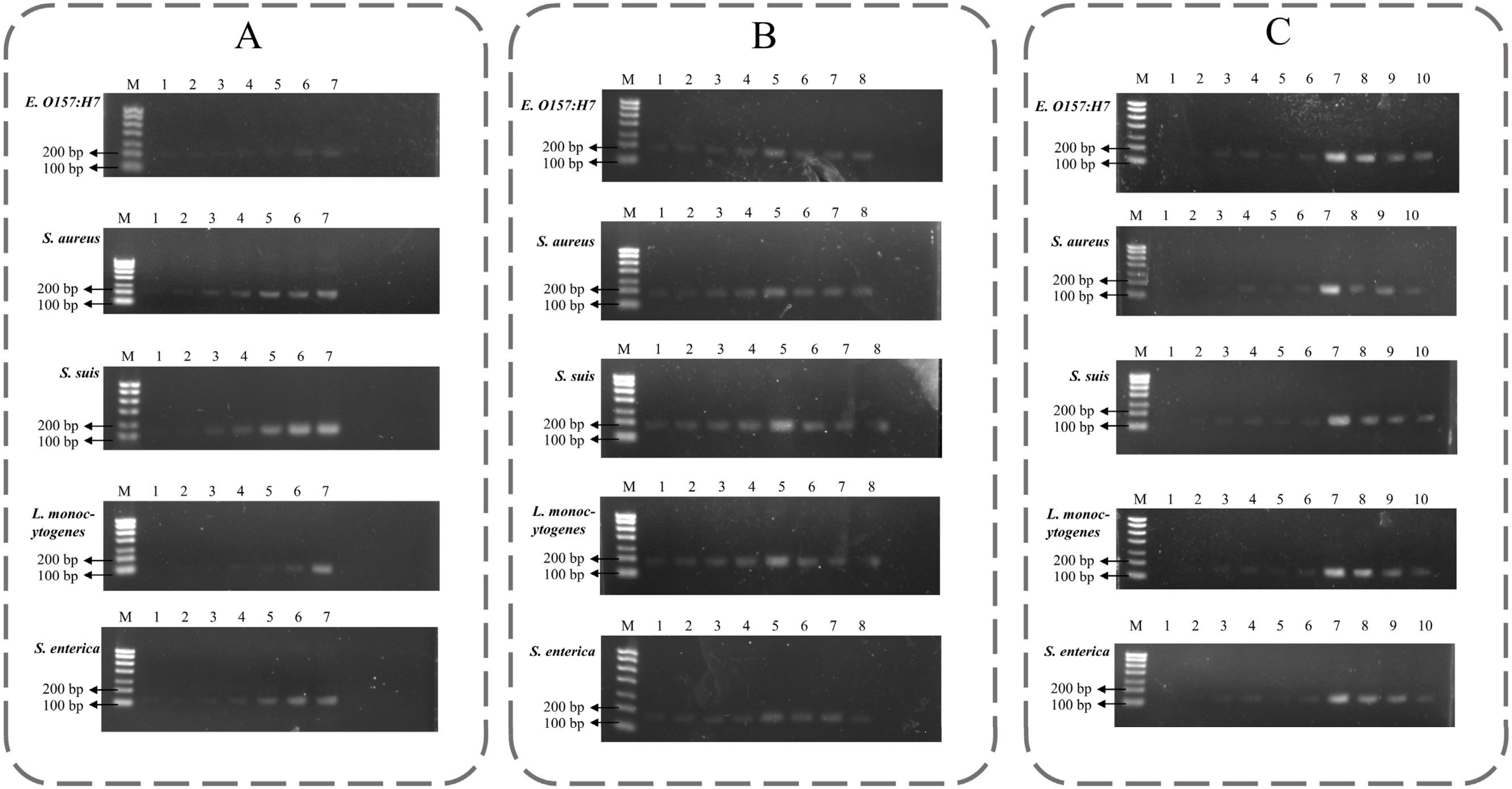

Figure S2. Agarose gel electrophoresis results for optimization of RPA amplification. (A) Primer concentration, (B) reaction temperature, (C) reaction time.

Table S1. Comments on five types of pathogens bacterial infection routes and symptoms

Disease	Bacteria	Associated food products	Route of infection	Symptoms	Comments	References
Leptospirosis	*Listeria monocytogenes*	Soft cheeses, pâté, milk, fried fish, and meat products	Skin or mucous membranes (eyes, nose, or mouth)	Septicemia, gastroenteritis, spontaneous abortion, meningitis, pneumonia	*Listeria monocytogenes* is considered to be pathogenic to humans. Although the global incidence of listeriosis is low, the disease has a very high hospitalization rate (over 95%) and a high mortality rate.	[1,2,3]
Bumblefoot/ foodborne disease	*Staphylococcus aureus*	Mice, juice, shrimp, milk, meat products	Consuming food or water contaminated with feces from infected animals or humans	Acne, osteomyelitis, endocarditis, respiratory tract infection, and septicemia	Zoonoses of *Staphylococcus aureus* are mainly associated with animal hosts (e.g. pigs, chickens), where methicillin-resistant *Staphylococcus aureus* (MRSA) is considered an emerging zoonotic pathogen of public health and veterinary importance.	[4,5,6,7]
Salmonellosis	*Salmonella enterica*	Pork products, poultry, seafood, milk, raw chicken, egg, shellfish	Eating or drinking inhaling, skin penetration, contaminated food	Fever, abdominal pain, headache, vomiting and sometimes septicemia	Most cases were caused by *Salmonella enterica* and *Salmonella typhimurium*. There are more than 10,000 cases of salmonellosis in the United States each year, resulting in more than 3,526 hospitalizations and 500 deaths.	[8,9,10,11]
Streptococcal meningitis syndrome (SMS)	*Streptococcus suis*	Meat products (Pigs cattle, sheep, goats)	Consumption of contaminated pig products or exposure to sick pigs	Meningitis, septicemia, pneumonia and arthritis	*Streptococcus suis* can cause serious clinical disease in pigs and humans, and there have been two large outbreaks of fatal human infections in China.	[12,13,14]
Foodborne disease	*Escherichia coli O157:H7*	Vegetables, drinking water, meat and dairy products	Livestock, drinking water, person-to-person, animal exposure	Diarrhea, vomiting, arthritis, meningitis, hemolytic uremic syndrome (HUS)	The *O157:H7* strain is consistently causing zoonotic foodborne and waterborne outbreaks. An estimated 73,480 cases of disease caused by *Escherichia coli O157:H7* infection annually in the US result in 2,168 hospitalizations and 61 deaths.	[15,16,17]

Table S2. Characteristics of RPA and other isothermal amplification technologies

Isothermal technique	Template	Primers	Temperature (°C)	Incubation time (min)	Multiple detection	References
RPA	DNA/RNA	2	37	15−20	Yes	[18,19]
RCA	DNA/RNA	1	37	60−240	No	[20,21]
LAMP	DNA	4-6	60-65	60	Yes	[22,23]
HAD	DNA	2	60-65	30−120	No	[24,25]
SDA	DNA	4	37	30	Yes	[26,27]

"RPA": Recombinase Protein Amplification; "RCA": Rolling circle Amplification; "LAMP": Loop-mediated isothermal Amplification Method; "HAD": Helicase-Dependent Amplification; "SDA": Strand Displacement Amplification.

Table S3. The sequences of primers used in this study

Target gene	Gene	The primer (5'−3')	Location (bp)	References
Listeria monocytogenes	*hlyA*	F: Cy5-CGATCACTCTGGAGGATACGTTGCTCAATT<br>R: digoxin-TTACCAGGCAAATAGATGGACGATGTGAAA	154	[28]
Staphylococcus aureus	*nuc*	F: FAM-CTTATAGGGATGGCTATCAGTAATGTTTCG<br>R: digoxin-CCACTTCTATTTACGCCGTTATCTGTTTGT	158	
Salmonella enterica	*fimY*	F: TAMRA-TATCAGATAAAACCTCCGCTATAACACAGT<br>R: digoxin-CTTTCCGATAAGCGAGGTTTGGAGGCTGAT	133	
Escherichia coli O157:H7	*rfbE*	F: biotin-TATCTGCAAGGTGATTCCTTGATGGTCTCA<br>R:digoxin-AGGCCAGTTACCATCCTCAGCTATAGGGTG	176	
Streptococcus suis	*gdh*	F: TET-ATTCATCAAACAATTATCAAAGGTAAATCCAC<br>R: digoxin-CTTCATTTACTACTAACATTCGGATTTTGCAA	147	/

F: forward primer; R:reverse primer; TAMRA: carboxytetramethylrhodamine; FAM: carboxy fluorescein; TET: tetrachlorofluorescein; Cy5: cyanine 5.

Table S4. Comparison of the quintuple RPA-EuNP-LFSBs with other biosensors

Method	Analyte	Food matrix	LOD ($CFU \cdot mL^{-1}$)	Reference
pNC-based strip biosensor	*Salmonella enteritidis*	Potable water, cole slaw cabbage salad, watermelon juice, purple cabbage and salad	10^2	[29]
Colloidal gold nanoparticle-based immunochromatographic test strip	*Staphylococcus aureus*	–	10^3	[30]
Electrochemical biosensors	*Listeria monocytogenes*	Lettuce, milk and ground beef	10^3	[31]
Two multianalyte Ab-based LFIA	*Escherichia coli O157*	Ground beef	10^5	[32]
Colloidal gold-based immunochromatographic assay	*Streptococcus suis*	–	10^6	[33]
SERS-based LF strip biosensor	*Listeria monocytogenes*<br>*Salmonella enterica*	Milk, chicken breast and beef	1.9×10^1<br>2.7×10^1	[34]
Pressed Paper-Based Dipstick	*Escherichia coli O157*<br>*S. typhimurium*	–	10^5<br>10^6	[35]
Phage display library technology	*Escherichia coli O157:H7*<br>*Listeria monocytogenes*	Cabbage	10^3<br>10^2	[36]
ME-biosensor	*Methicillin-resistant Staphylococcus aureus*<br>*Listeria monocytogenes*<br>*Escherichia coli O157:H7*<br>*Salmonella Typhimurium*	–	62.5 ± 11.0<br>56.2 ± 11.8<br>59.4 ± 10.5<br>56.0 ± 10.7	[37]
Quintuple RPA-EuNP-LFSBs	*Listeria monocytogenes*<br>*Staphylococcus aureus*<br>*Streptococcus suis*<br>*Salmonella enterica*<br>*Escherichia coli O157:H7*	Chicken, pork, beef, lamb, duck and milk	1.5×10^1<br>3.2×10^1<br>2.2×10^1<br>1.9×10^1<br>1.7×10^1	This study

"LOD": limit of detection; "SERS": surface-enhanced Raman scattering; "LF": lateral flow;

“AuNPs”: gold nanoparticles; “ME": magnetoelastic.

Table S5. The artificially contamination of individual strains in the samples.

	Listeria monocytogenes				*Staphylococcus aureus*				*Streptococcus suis*				*Salmonella enterica*				*Escherichia coli O157:H7*			
Samples (n=3 Each)	Inoculation Level (CFU/mL or CFU/g)	Quintuple RPA-EuNP-LFSBs DetectedConcentration (CFU/mL or CFU/g)	Recovery (%)	culture method	Inoculation Level (CFU/mL or CFU/g)	Quintuple RPA-EuNP-LFSBs DetectedConcentration (CFU/mL or CFU/g)	Recovery (%)	culture method	Inoculation Level (CFU/mL or CFU/g)	Quintuple RPA-EuNP-LFSBs DetectedConcentration (CFU/mL or CFU/g)	Recovery (%)	culture method	Inoculation Level (CFU/mL or CFU/g)	Quintuple RPA-EuNP-LFSBs DetectedConcentration (CFU/mL or CFU/g)	Recovery (%)	culture method	Inoculation Level (CFU/mL or CFU/g)	Quintuple RPA-EuNP-LFSBs DetectedConcentration (CFU/mL or CFU/g)	Recovery (%)	culture method
Chicken	1.9×10^4	1.86×10^4	97.9	+	3.8×10^4	3.66×10^4	96.3	+	2.4×10^4	2.33×10^4	97.1	+	2.2×10^4	2.09×10^4	95.0	+	1.9×10^4	1.85×10^4	97.4	+
	1.9×10^3	1.92×10^3	101.1	+	3.8×10^3	3.72×10^3	97.9	+	2.4×10^3	2.36×10^3	98.3	+	2.2×10^3	2.03×10^3	92.3	+	1.9×10^3	1.88×10^3	98.9	+
	1.9×10^2	1.77×10^2	93.2	+	3.8×10^2	3.76×10^2	98.9	+	2.4×10^2	2.24×10^2	93.3	+	2.2×10^2	2.13×10^2	96.8	+	1.9×10^2	1.82×10^2	95.8	+
	1.9×10^1	1.83×10^1	96.3	−	3.8×10^1	3.66×10^1	96.3	−	2.4×10^1	2.31×10^1	96.3	−	2.2×10^1	2.03×10^1	92.3	−	1.9×10^1	1.75×10^1	92.1	−
Pork	1.9×10^4	1.87×10^4	98.4	+	3.8×10^4	3.88×10^4	100.0	+	2.4×10^4	2.32×10^4	96.7	+	2.2×10^4	2.13×10^4	96.8	+	1.9×10^4	1.81×10^4	95.3	+
	1.9×10^3	1.88×10^3	98.9	+	3.8×10^3	3.78×10^3	99.5	+	2.4×10^3	2.40×10^3	100.0	+	2.2×10^3	2.10×10^3	95.5	+	1.9×10^3	1.84×10^3	96.8	+
	1.9×10^2	1.92×10^2	101.1	+	3.8×10^2	3.76×10^2	98.9	+	2.4×10^2	2.23×10^2	92.9	+	2.2×10^2	2.15×10^2	97.7	+	1.9×10^2	1.79×10^2	94.2	+
	1.9×10^1	1.87×10^1	98.4	−	3.8×10^1	3.67×10^1	96.6	−	2.4×10^1	2.27×10^1	94.6	−	2.2×10^1	2.06×10^1	93.6	−	1.9×10^1	1.88×10^1	98.9	−
Beef	1.9×10^4	1.87×10^4	98.4	+	3.8×10^4	3.81×10^4	100.3	+	2.4×10^4	2.26×10^4	94.2	+	2.2×10^4	2.16×10^4	98.2	+	1.9×10^4	1.87×10^4	98.4	+
	1.9×10^3	1.88×10^3	98.9	+	3.8×10^3	3.77×10^3	99.2	+	2.4×10^3	2.33×10^3	97.1	+	2.2×10^3	2.15×10^3	97.7	+	1.9×10^3	1.85×10^3	97.4	+
	1.9×10^2	1.74×10^2	91.6	+	3.8×10^2	3.75×10^2	98.7	+	2.4×10^2	2.35×10^2	97.9	+	2.2×10^2	2.08×10^2	94.5	+	1.9×10^2	1.89×10^2	99.5	+
	1.9×10^1	1.75×10^1	92.1	−	3.8×10^1	3.72×10^1	97.9	−	2.4×10^1	2.33×10^1	97.1	−	2.2×10^1	2.05×10^1	93.2	−	1.9×10^1	1.83×10^1	96.3	−
Lamb	1.9×10^4	1.77×10^4	93.2	+	3.8×10^4	3.73×10^4	98.2	+	2.4×10^4	2.36×10^4	98.3	+	2.2×10^4	2.13×10^4	96.8	+	1.9×10^4	1.91×10^4	100.5	+
	1.9×10^3	1.88×10^3	98.9	+	3.8×10^3	3.77×10^3	99.2	+	2.4×10^3	2.30×10^3	95.8	+	2.2×10^3	2.16×10^3	98.2	+	1.9×10^3	1.90×10^3	100.0	+

	1.9×10^{2}	1.82×10^{2}	95.8	+	3.8×10^{2}	3.71×10^{2}	97.6	+	2.4×10^{2}	2.21×10^{2}	92.1	+	2.2×10^{2}	2.19×10^{2}	99.5	+	1.9×10^{2}	1.87×10^{2}	98.4	+
	1.9×10^{1}	1.83×10^{1}	96.3	–	3.8×10^{1}	3.70×10^{1}	97.4	–	2.4×10^{1}	2.3×10^{1}	95.8	–	2.2×10^{1}	2.05×10^{1}	93.2	–	1.9×10^{1}	1.85×10^{1}	97.4	–
Duck	1.9×10^{4}	1.85×10^{4}	97.4	+	3.8×10^{4}	3.76×10^{4}	98.9	+	2.4×10^{4}	2.27×10^{4}	94.6	+	2.2×10^{4}	2.19×10^{4}	99.5	+	1.9×10^{4}	1.89×10^{4}	99.5	+
	1.9×10^{3}	1.74×10^{3}	91.6	+	3.8×10^{3}	3.77×10^{3}	99.2	+	2.4×10^{3}	2.26×10^{3}	94.2	+	2.2×10^{3}	2.17×10^{3}	98.6	+	1.9×10^{3}	1.86×10^{3}	97.9	+
	1.9×10^{2}	1.78×10^{2}	93.7	+	3.8×10^{2}	3.78×10^{2}	99.5	+	2.4×10^{2}	2.31×10^{2}	96.3	+	2.2×10^{2}	2.13×10^{2}	96.8	+	1.9×10^{2}	1.81×10^{2}	95.3	+
	1.9×10^{1}	1.76×10^{1}	92.6	–	3.8×10^{1}	3.71×10^{1}	97.6	–	2.4×10^{1}	2.35×10^{1}	97.9	–	2.2×10^{1}	2.08×10^{1}	94.5	–	1.9×10^{1}	1.79×10^{1}	94.2	–
Milk	1.9×10^{4}	1.88×10^{4}	98.9	+	3.8×10^{4}	3.79×10^{4}	99.7	+	2.4×10^{4}	2.38×10^{4}	99.2	+	2.2×10^{4}	2.1×10^{4}	95.5	+	1.9×10^{4}	1.89×10^{4}	99.5	+
	1.9×10^{3}	1.87×10^{3}	98.4	+	3.8×10^{3}	3.77×10^{3}	99.2	+	2.4×10^{3}	2.37×10^{3}	98.8	+	2.2×10^{3}	2.14×10^{3}	97.3	+	1.9×10^{3}	1.87×10^{3}	98.4	+
	1.9×10^{2}	1.79×10^{2}	94.2	+	3.8×10^{2}	3.75×10^{2}	98.7	+	2.4×10^{2}	2.35×10^{2}	97.9	+	2.2×10^{2}	2.17×10^{2}	98.6	+	1.9×10^{2}	1.81×10^{2}	95.3	+
	1.9×10^{1}	1.75×10^{1}	92.1	–	3.8×10^{1}	3.72×10^{1}	97.9	–	2.4×10^{1}	2.32×10^{1}	96.7	–	2.2×10^{1}	2.09×10^{1}	95.0	–	1.9×10^{1}	1.84×10^{1}	96.8	–

"culture method": the bacteriological analytical manual (BAM) or the national standard (GB/T 19915.2-2005).

Table S6. Five target bacteria co-existed in the food samples.

Samples (n=3 Each)	Strains	Inoculation Level* (CFU/mL or CFU/g)	Quintuple RPA-EuNP-LFSBs Detected Concentration (CFU/mL or CFU/g)	Recoveries (%)	culture method
Chicken	*Streptococcus suis*	2.2×10^4	2.07×10^4	90.6−97.8	+
	Staphylococcus aureus	3.2×10^4	3.13×10^4		
	Salmonella enterica	1.9×10^4	1.79×10^4		
	Escherichia coli O157:H7	1.7×10^4	1.54×10^4		
	Listeria monocytogenes	1.5×10^4	1.41×10^4		
	Streptococcus suis	2.2×10^3	2.04×10^3	92.7−100.9	+
	Staphylococcus aureus	3.2×10^3	3.23×10^3		
	Salmonella enterica	1.9×10^3	1.86×10^3		
	Escherichia coli O157:H7	1.7×10^3	1.63×10^3		
	Listeria monocytogenes	1.5×10^3	1.45×10^3		
	Streptococcus suis	2.2×10^2	2.19×10^2	93.4−101.6	+
	Staphylococcus aureus	3.2×10^2	2.99×10^2		
	Salmonella enterica	1.9×10^2	1.93×10^2		
	Escherichia coli O157:H7	1.7×10^2	1.71×10^2		
	Listeria monocytogenes	1.5×10^2	1.43×10^2		
	Streptococcus suis	2.2×10^1	2.16×10^1	91.6−100.6	−
	Staphylococcus aureus	3.2×10^1	3.16×10^1		
	Salmonella enterica	1.9×10^1	1.74×10^1		
	Escherichia coli O157:H7	1.7×10^1	1.71×10^1		
	Listeria monocytogenes	1.5×10^1	1.41×10^1		
Pork	*Streptococcus suis*	2.2×10^4	2.17×10^4	91.6−100.5	+
	Staphylococcus aureus	3.2×10^4	2.93×10^4		
	Salmonella enterica	1.9×10^4	1.91×10^4		
	Escherichia coli O157:H7	1.7×10^4	1.69×10^4		
	Listeria monocytogenes	1.5×10^4	1.44×10^4		
	Streptococcus suis	2.2×10^3	2.20×10^3	91.1−100.0	+
	Staphylococcus aureus	3.2×10^3	3.19×10^3		
	Salmonella enterica	1.9×10^3	1.73×10^3		
	Escherichia coli O157:H7	1.7×10^3	1.67×10^3		
	Listeria monocytogenes	1.5×10^3	1.45×10^3		
	Streptococcus suis	2.2×10^2	2.16×10^2	95.3−100.0	+
	Staphylococcus aureus	3.2×10^2	3.20×10^2		
	Salmonella enterica	1.9×10^2	1.88×10^2		
	Escherichia coli O157:H7	1.7×10^2	1.68×10^2		
	Listeria monocytogenes	1.5×10^2	1.43×10^2		
	Streptococcus suis	2.2×10^1	2.13×10^1	96.5−101.6	−

	Staphylococcus aureus	3.2×10^1	3.23×10^1		
	Salmonella enterica	1.9×10^1	1.93×10^1		
	Escherichia coli O157:H7	1.7×10^1	1.64×10^1		
	Listeria monocytogenes	1.5×10^1	1.48×10^1		
	Streptococcus suis	2.2×10^4	2.04×10^4		
	Staphylococcus aureus	3.2×10^4	2.96×10^4		
	Salmonella enterica	1.9×10^4	1.84×10^4	92.5−97.6	+
	Escherichia coli O157:H7	1.7×10^4	1.66×10^4		
	Listeria monocytogenes	1.5×10^4	1.44×10^4		
	Streptococcus suis	2.2×10^3	2.07×10^3		
	Staphylococcus aureus	3.2×10^3	3.24×10^3		
	Salmonella enterica	1.9×10^3	1.81×10^3	94.1−101.3	+
	Escherichia coli O157:H7	1.7×10^3	1.71×10^3		
Beef	*Listeria monocytogenes*	1.5×10^3	1.43×10^3		
	Streptococcus suis	2.2×10^2	2.16×10^2		
	Staphylococcus aureus	3.2×10^2	2.96×10^2		
	Salmonella enterica	1.9×10^2	1.79×10^2	90.7−98.2	+
	Escherichia coli O157:H7	1.7×10^2	1.67×10^2		
	Listeria monocytogenes	1.5×10^2	1.36×10^2		
	Streptococcus suis	2.2×10^1	2.01×10^1		
	Staphylococcus aureus	3.2×10^1	3.21×10^1		
	Salmonella enterica	1.9×10^1	1.80×10^1	91.4−101.2	−
	Escherichia coli O157:H7	1.7×10^1	1.72×10^1		
	Listeria monocytogenes	1.5×10^1	1.43×10^1		
	Streptococcus suis	2.2×10^4	2.03×10^4		
	Staphylococcus aureus	3.2×10^4	3.25×10^4		
	Salmonella enterica	1.9×10^4	1.78×10^4	92.3−101.6	+
	Escherichia coli O157:H7	1.7×10^4	1.72×10^4		
	Listeria monocytogenes	1.5×10^4	1.45×10^4		
	Streptococcus suis	2.2×10^3	2.08×10^3		
	Staphylococcus aureus	3.2×10^3	3.24×10^3		
	Salmonella enterica	1.9×10^3	1.92×10^3	92.7−101.3	+
Lamb	*Escherichia coli O157:H7*	1.7×10^3	1.68×10^3		
	Listeria monocytogenes	1.5×10^3	1.39×10^3		
	Streptococcus suis	2.2×10^2	2.11×10^2		
	Staphylococcus aureus	3.2×10^2	3.10×10^2		
	Salmonella enterica	1.9×10^2	1.92×10^2	94.7−101.1	+
	Escherichia coli O157:H7	1.7×10^2	1.69×10^2		
	Listeria monocytogenes	1.5×10^2	1.42×10^2		
	Streptococcus suis	2.2×10^1	2.13×10^1		
	Staphylococcus aureus	3.2×10^1	3.10×10^1	94.7−101.1	−
	Salmonella enterica	1.9×10^1	1.92×10^1		

	Escherichia coli O157:H7	1.7×10^{1}	1.71×10^{1}		
	Listeria monocytogenes	1.5×10^{1}	1.42×10^{1}		
	Streptococcus suis	2.2×10^{4}	2.19×10^{4}		
	Staphylococcus aureus	3.2×10^{4}	3.02×10^{4}		
	Salmonella enterica	1.9×10^{4}	1.77×10^{4}	94.5−101.2	+
	Escherichia coli O157:H7	1.7×10^{4}	1.72×10^{4}		
	Listeria monocytogenes	1.5×10^{4}	1.47×10^{4}		
	Streptococcus suis	2.2×10^{3}	2.08×10^{3}		
	Staphylococcus aureus	3.2×10^{3}	3.10×10^{3}		
	Salmonella enterica	1.9×10^{3}	1.86×10^{3}	94.5−100.7	+
	Escherichia coli O157:H7	1.7×10^{3}	1.69×10^{3}		
Duck	*Listeria monocytogenes*	1.5×10^{3}	1.51×10^{3}		
	Streptococcus suis	2.2×10^{2}	2.18×10^{2}		
	Staphylococcus aureus	3.2×10^{2}	3.18×10^{2}		
	Salmonella enterica	1.9×10^{2}	1.79×10^{2}	94.2−101.3	+
	Escherichia coli O157:H7	1.7×10^{2}	1.68×10^{2}		
	Listeria monocytogenes	1.5×10^{2}	1.52×10^{2}		
	Streptococcus suis	2.2×10^{1}	2.12×10^{1}		
	Staphylococcus aureus	3.2×10^{1}	3.05×10^{1}		
	Salmonella enterica	1.9×10^{1}	1.90×10^{1}	95.3−101.2	−
	Escherichia coli O157:H7	1.7×10^{1}	1.72×10^{1}		
	Listeria monocytogenes	1.5×10^{1}	1.51×10^{1}		
	Streptococcus suis	2.2×10^{4}	2.11×10^{4}		
	Staphylococcus aureus	3.2×10^{4}	3.22×10^{4}		
	Salmonella enterica	1.9×10^{4}	1.82×10^{4}	95.3−100.6	+
	Escherichia coli O157:H7	1.7×10^{4}	1.69×10^{4}		
	Listeria monocytogenes	1.5×10^{4}	1.43×10^{4}		
	Streptococcus suis	2.2×10^{3}	2.13×10^{3}		
	Staphylococcus aureus	3.2×10^{3}	3.22×10^{3}		
	Salmonella enterica	1.9×10^{3}	1.93×10^{3}	96.8−101.6	+
	Escherichia coli O157:H7	1.7×10^{3}	1.66×10^{3}		
	Listeria monocytogenes	1.5×10^{3}	1.47×10^{3}		
Milk	*Streptococcus suis*	2.2×10^{2}	2.22×10^{2}		
	Staphylococcus aureus	3.2×10^{2}	3.12×10^{2}		
	Salmonella enterica	1.9×10^{2}	1.91×10^{2}	96.0−100.9	+
	Escherichia coli O157:H7	1.7×10^{2}	1.67×10^{2}		
	Listeria monocytogenes	1.5×10^{2}	1.44×10^{2}		
	Streptococcus suis	2.2×10^{1}	2.23×10^{1}		
	Staphylococcus aureus	3.2×10^{1}	3.22×10^{1}		
	Salmonella enterica	1.9×10^{1}	1.79×10^{1}	94.2−101.4	−
	Escherichia coli O157:H7	1.7×10^{1}	1.63×10^{1}		
	Listeria monocytogenes	1.5×10^{1}	1.51×10^{1}		

"*": Equal volume factor mixing at different concentration levels before and after mixing; "culture method": the bacteriological analytical manual (BAM) or the national standard (GB/T 19915.2-2005).

References

[1] Sant'Ana, A.S.; Franco, B.; Schaffner, D.W. Risk of infection with *Salmonella* and *Listeria monocytogenes* due to consumption of ready-to-eat leafy vegetables in brazil. *Food Control* **2014**, *42*, 1–8.

[2] Carvalho, F.; Sousa, S.; Cabanes, D. How listeria monocytogenes organizes its surface for virulence. *Front. Cell. Infect. Microbiol*. **2014**, *4*, 00048.

[3] Scallan, E.; Hoekstra, B.M.; Angulo, F.J.; Tauxe, R.V.; Widdowson, M.A.; Roy, S.L.; Jones, J.L.; Griffin, P.M. Foodborne illness acquired in the United States—Major pathogens. *Emerg. Infect. Dis*. **2011**, *17*, 7–15.

[4] Aklilu, E.; Zunita Z.; Hassan, L.; Chen, H. Phenotypic and genotypic characterization of methicillin-resistant *Staphylococcus aureus* (MRSA) isolated from dogs and cats at University Veterinary Hospital, Universiti Putra Malaysia. *Trop Biomed*. **2010**;*27*, 483–492.

[5] Fitzgerald, J.R. Livestock-associated staphylococcus aureus: Origin, evolution and public health threat. *Trends in microbiology* **2012**, *20*, 192-198.

[6] Arunachalam, K.; Pandurangan, P.; Shi, C.; Lagoa, R. Regulation of *Staphylococcus aureus* virulence and application of Nanotherapeutics to Eradicate *S. aureus* infection. *Pharmaceutics* **2023**, *15*. 310.

[7] Algammal, A.M.; Hetta, H.F.; Elkelish, A.; Alkhalifah, D.H.H.; Hozzein, W.N.; Batiha, G.E.; Nahhas, N.; Mabrok, M.A. Methicillin-resistant *Staphylococcus aureus* (MRSA): one Health perspective approach to the bacterium epidemiology, virulence factors, antibiotic-resistance, and zoonotic impact. *Infection and drug resist*. **2020**, *13*, 3255-3265.

[8] Ferrari R.G.; Rosario D.K.A.; Cunha-Neto A.; Mano S.B.; Figueiredo E.E.S.; Conte-Junior C.A. Worldwide epidemiology of *Salmonella* serovars in animal-based foods: A meta-analysis. *Appl Environ Microbiol*. **2019**, *85*, e00591-19.

[9] Antunes, P.; Novais, C.; Peixe, L. Food-to-humans bacterial transmission. *Microbiol spectr*. **2020**, 8.

[10] Stevens, M.P.; Humphrey, T.J.; Maskell, D.J. Molecular insights into farm animal and zoonotic *Salmonella* infections. *Philos Trans R Soc Lond B Biol Sci.* **2009**, *364*,

2709-2723.

[11] Centers for Disease Control and Prevention (CDC) *Salmonella*. Available online: https://www.cdc.gov/salmonella/index.html (accessed on 19th January 2023)

[12] Marois, C.; Bougeard, S.; Gottschalk, M.; Kobisch, M. Multiplex PCR assay for detection of *Streptococcus suis* species and serotypes 2 and 1/2 in tonsils of live and dead pigs. *J Clin Microbiol*. **2004**, *42*, 3169-3175.

[13] Segura M. *Streptococcus suis*: An emerging human threat. *J. Infect. Dis.* **2009**, *199*, 97-107.

[14] Wang, Y.; Wang, Y.X.; Sun, L.; Grenier, D.; Yi, L. *Streptococcus suis* biofilm: Regulation, drug-resistance mechanisms, and disinfection strategies. *Appl Microbiol Biotechnol*. **2018**, *102*, 9121-9129.

[15] Chekabab, S.M.; Paquin-Veillette, J.; Dozois, C.M.; Harel, J. The ecological habitat and transmission of *Escherichia coli o157:H7*. *FEMS microbio lett*. **2013**, *341*, 1-12.

[16] Rangel, J.M.; Sparling, P.H.; Crowe, C.; Griffin, P.M.; Swerdlow, D.L. Epidemiology of *Escherichia coli O157:H7* outbreaks, united states, 1982-2002. *Emerg Infect Dis*. **2005**, *11*, 603-609.

[17] Mead, P.S.; Slutsker, L.; Dietz, V.; McCaig, L.F.; Bresee, J.S.; Shapiro, C.; Griffin, P.M.; Tauxe, R.V. Food-related illness and death in the United States. *Emerg Infect Dis*. **1999**, *5*, 607-625.

[18] Lillis, L.; Siverson, J.; Lee A.; Cantera, J.; Parker, M.; Piepenburg, O.; Lehman, D.A.; Boyle, D.S. Factors influencing recombinase polymerase amplification (RPA) assay outcomes at point of care. *Mol Cell Probes*. **2016**, *30*, 74-78.

[19] Lobato, I.M.; O'Sullivan, C.K. Recombinase polymerase amplification: Basics, applications and recent advances. *Trends Analyt Chem.* **2018**, *98*, 19-35.

[20] Rodrigues, A.M.; Najafzadeh, M.J.; de Hoog, G.S.; de Camargo, Z.P. Rapid identification of emerging human-pathogenic *Sporothrix* species with rolling circle amplification. *Front Microbiol* **2015**, *6*, 1385.

[21] Mao, Y.; Liu, M.; Tram, K.; Gu, J.; Salena, B.J.; Jiang, Y.; Li, Y. Optimal DNA templates for rolling circle amplification revealed by in vitro selection. *Chemistry*. **2015**,

21, 8069-8074.

[22] Jaroenram, W.; Kiatpathomchai, W.; Flegel, T.W. Rapid and sensitive detection of white spot syndrome virus by loop-mediated isothermal amplification combined with a lateral flow dipstick. *Mol Cell Probes*. **2009**, *23*, 65-70.

[23] Luo, J.; Vogel, R.F.; Niessen, L. Development and application of a loop-mediated isothermal amplification assay for rapid identification of aflatoxigenic molds and their detection in food samples. *Int J Food Microbiol*. **2012**, *159*, 214-224.

[24] Kolm, C.; Martzy, R.; Führer, M.; Mach, R.L.; Krska, R.; Baumgartner, S.; Farnleitner, A.H.; Reischer, G.H. Detection of a microbial source tracking marker by isothermal helicase-dependent amplification and a nucleic acid lateral-flow strip test. *Sci Rep*. **2019**, *9*, 393.

[25] Liu, M.; Li, C.C.; Luo, X.; Ma, F.; Zhang, C.Y. 5-hydroxymethylcytosine glucosylation-triggered helicase-dependent amplification-based fluorescent biosensor for sensitive detection of β-glucosyltransferase with zero background signal. *Anal Chem*. **2020**, *92*, 16307-16313.

[26] Walker, G.T.; Fraiser, M.S.; Schram, J.L.; Little, M.C.; Nadeau, J.G.; Malinowski, D.P. Strand displacement amplification--an isothermal, in vitro DNA amplification technique. *Nucleic Acids Res*. **1992**, *20*, 1691-1696.

[27] Tang, S.; Liu, Y.; Yan, Y.; Serge, Y.O.; Zhou, X.; Xiang, X.; Ma, C. A highly sensitive method for the detection of alkaline phosphatase based on thioflavin t/g-quadruplex and strand displacement amplification. *Microchem. J.* **2022**, *181*, 107655.

[28] Jin, B.; Ma, B.; Li, J.; Hong, Y.; Zhang, M. Simultaneous detection of five foodborne pathogens using a mini automatic nucleic acid extractor combined with recombinase polymerase amplification and lateral flow immunoassay. *Microorganisms* **2022**, *10*, 1352.

[29] Wang, Z.; Yao, X.; Wang, R.; Ji, Y.; Yue, T.; Sun, J.; Li, T.; Wang, J.; Zhang, D. Label-free strip sensor based on surface positively charged nitrogen-rich carbon nanoparticles for rapid detection of *Salmonella enteritidis*. *Biosensors & bioelectronics* **2019**, *132*, 360-367.

[30] Niu, K.; Zheng, X.; Huang, C.; Xul, K.; Zhi, Y.; Shen, H.; Jia, N. A colloidal gold

nanoparticle-based immunochromatographic test strip for rapid and convenient detection of S*taphylococcus aureus*. *J Nanosci Nanotechnol*. **2014**, *14*, 5151-5156.
[31] Kanayeva, D.A.; Wang, R.; Rhoads, D.; Erf, G.F.; Slavik, M.F.; Tung, S.; Li, Y. Efficient separation and sensitive detection of listeria monocytogenes using an impedance immunosensor based on magnetic nanoparticles, a microfluidic chip, and an interdigitated microelectrode. *J Food Prot*. **2012**, *75*, 1951-1959.
[32] Wang, J.; Katani, R.; Li, L.; Hegde, N.; Roberts, E.L.; Kapur, V.; DebRoy, C. Rapid detection of *Escherichia Coli O157* and *Shiga* toxins by lateral flow immunoassays. *Toxins* **2016**, *8*, 92.
[33] Ju, Y.; Hao, H.J.; Xiong, G.H.; Geng, H.R.; Zheng, Y.L.; Wang, J.; Cao, Y.; Yang, Y.H.; Cai, X.H.; Jiang, Y.Q. Development of colloidal gold-based immunochromatographic assay for rapid detection of *Streptococcus suis* serotype 2. *Vet Immunol Immunopathol.* **2010**, *133*, 207-211.
[34] Liu, H.B.; Du, X.J.; Zang, Y.X.; Li, P.; Wang, S. Sers-based lateral flow strip biosensor for simultaneous detection of listeria monocytogenes and *Salmonella enterica* serotype enteritidis. *J Agric Food Chem.* **2017**, *65*, 10290-10299.
[35] Park, J.; Shin, J.H.; Park, J.K. Pressed paper-based dipstick for detection of foodborne pathogens with multistep reactions. *Anal Chem*. **2016**, *88*, 3781-3788.
[36] Li, L.; Zhang, H.; Song, D.; Xu, K.; Zheng, Y.; Xiao, H.; Liu, Y.; Li, J.; Song, X. Simultaneous detection of three zoonotic pathogens based on phage display peptide and multicolor quantum dots. *Anal Biochem*. **2020**, *608*, 113854.
[37] Hiremath, N.; Chin, B.A.; Park, M.K. Effect of competing foodborne pathogens on the selectivity and binding kinetics of a lytic phage for methicillin-resistant staphylococcus aureus detection. *J. Electrochem. Soc.* **2017**, 164.
